# Perception and Demands of Pregnant and Breastfeeding Women Regarding Their Role as Participants in Environmental Research Studies

**DOI:** 10.3390/ijerph18084149

**Published:** 2021-04-14

**Authors:** Miguel Company-Morales, Eva Zafra Aparici, Lina Casadó, Cristina Alarcón Montenegro, Juan Pedro Arrebola

**Affiliations:** 1 Seron Primary Care Center, Northern Almería Integrated Healthcare Area, 04600 Huercal-Overa, Almería, Spain; 2 Department of Nursing, Physiotherapy and Medicine, University of Almería, 04120 La Cañada, Almería, Spain; 3 Department of Anthropology, Philosophy and Social Work, University Rovira i Virgili, 43003 Tarragona, Tarragona, Spain; eva.zafra@urv.cat; 4 Department of Nursing, Medical Anthropology Research Centre (MARC), University Rovira i Virgili, 43003 Tarragona, Tarragona, Spain; linacristina.casado@urv.cat; 5 Antequera Hospital, Northern Málaga Integrated Healthcare Area, 29200 Antequera, Malaga, Spain; cristinaalarconm84@gmail.com; 6 Department of Preventive Medicine and Public Health, University of Granada, 18071 Granada, Granada, Spain; jparrebola@ugr.es; 7 Instituto de Investigación Biosanitaria de Granada (ibs.GRANADA), 18012 Granada, Granada, Spain; 8 CIBER de Epidemiología y Salud Pública (CIBERESP), 28029 Madrid, Granada, Spain

**Keywords:** pregnancy, breastfeeding, vulnerable population, environmental risk, qualitative research

## Abstract

A significant proportion of scientific studies consider pregnant and breastfeeding women as vulnerable subjects. The objective of this study was to analyse the perception of pregnant and breastfeeding women regarding their participation in environmental research studies. Our work is a descriptive and interpretative observational study that has been developed under the qualitative research paradigm following a phenomenological and ethnographic perspective. The study involved 173 women selected intentionally in two Spanish autonomous communities. To obtain the primary data, we relied upon 111 interviews, four focused ethnographies and eight focus groups. The data encoding and analysis was carried out with the help of NVivo 12 software (QSR International, Boston, MA, USA). We evidenced the need of pregnant and breastfeeding women for more detailed and accurate information on the risk of environmental pollutant exposure during their crucial life stage. In addition, these women claimed for a more participatory role in research studies. Pregnant and breastfeeding women in Spain ask for greater interaction with researchers and propose a dialogical relationship between valid partners. We conclude that our pregnant and breastfeeding women claim more research focused on their collective, as well as clearer, more accessible and structured information on the risks of exposure to environmental contaminants. In addition, they do not want to simply be informants; rather, they ask to be active and empowered members by providing their opinions and arguments throughout the research process.

## 1. Introduction

The technological development in industrial, chemical and food production over the past 50 years has caused us to be globally exposed to different synthetic chemical compounds that have health and environmental effects [1,2,3,4,5,6,7,8]. There is growing evidence that continued exposure to low doses of pollutants, as occurs in most of the general population, could increase the risk of developing various chronic pathologies, such as cancer, cardiovascular disease or diabetes [9]. On the other hand, critical exposure windows have been described throughout human life, during which the individual would be much more vulnerable to the effects of these contaminants. These include pregnancy and lactation, as these exposures could also affect the health of the offspring. In fact, exposure to various contaminants during pregnancy has been associated with alterations in fetal growth, newborn weight, asthma, risk of pre-term birth or alterations in neurodevelopment, among others. For this reason, pregnant and breastfeeding women have traditionally been considered vulnerable subjects for scientific studies [10,11,12,13,14].

Despite the abovementioned evidences, results from observational studies in critical exposure windows remain controversial [15], hampering the result dissemination in the general population, particularly in highly vulnerable subgroups, such as pregnant women [16]. This lack of homogeneous information on environmental risks, along with the sense of vulnerability that frequently accompanies these periods of life, can become a source of stress for women [17]. These stressors include environmental risks related to potential chemical contamination of food [10,11,12,13,14]. Pérez-Padilla et al. [16] pointed out that the presence of stressors may be nuanced by the mother’s coping ability, so that empowerment strategies can play a relevant role when a stressful response appears or not, and that these might have greater or lesser intensity. Indeed, many research studies agree that better coping during pregnancy has beneficial effects not only on the mother, but also on the fetus, as it helps to decrease anxiety and thereby decrease complications during the pregnancy and the birth [18,19]. In addition, the risk of perinatal and postpartum depression is reduced, and the mother’s emotional health and stress management improves [17,18,19].

The emotional perception of insecurity and the risk associated with toxic chemical exposure, and its incorporation through the act of feeding, does not always coincide with the real dangers; rather, it is constructed on the basis of trust/distrust as a relational attitude that, according to Lomnitz [20] is imbued with order, cultural (reciprocity), physical and economic variables.

In previous works, our team noted that women at this stage of their life cycle are more sensitive to messages regarding the benefits of healthy eating [21]. However, at the same time, women may also feel greater uncertainty about actions that benefit or harm their health and that of their unborn child.

The construction of the pregnant woman as a subject of risk and vulnerability [22,23,24] sometimes makes her over-dependent on medical services and controls, and on many occasions leads to her being involved in research to which she is invited, assigning her—implicitly—the role of “passive subject” without the possibility of participation. Even though the current trend in obstetric and gynaecological practice and follow-up is to demedicalize and depathologize pregnancy and childbirth, the truth is that meticulous medical, ultrasound and analytical control is still being exercised over the woman and the fetus [25,26] to avoid potential risks.

As Toledo et al. point out [27], the new care models propose a scenario in which patients and users cease to be passive receivers of care, and instead are oriented towards making them active in their own health prevention and self-management [28]. For this to occur, Krucien et al. [29] point out that empowerment is needed as a tool to change and reorientate health systems. Likewise, UNWomen [30] indicates that the challenge for maternal and child health (one of the World Health Organization’s 2020 Objectives and also one of the Agenda 2030 Sustainable Development Objectives) [31,32] is to educate the population about health and to empower the participants themselves, making them responsible for their own health and able to transform their environment and community.

In recent years, numerous initiatives have been developed, both conceptually and practically, to empower health service users, moving away from paternalistic conceptions of the relationships with health professionals, and focusing on more horizontal and collaborative models and, therefore, on an integrated care [33]. In many cases, empowerment may include participation in collective processes (*EU Joint Action on Patient Safety and Quality of Care*, cited in the EPF, 2015) [34]; or people-centered care with shared decision-making between patients and healthcare professionals [35]. Of course, this also includes participatory researches with close interactions between health researchers and the participants. For this reason, food risk analysis in pregnant and breastfeeding women, empowerment and self-management are particularly relevant in our research because they allow us to deepen our understanding of women’s experiences [36,37]. For these reasons, social studies are warranted on the narratives of the perceptions and experiences of pregnant and breastfeeding women. These studies can help to understand the perception of the process of chemical exposure through food and how the participation in researches in the topic is lived and interpreted, as well as which are the main support requirements of the women and those they care for [38,39,40]. The narratives promote empowerment in the development of capacities, resources and skills related to feeding care and self-care in pregnant and breastfeeding women, as well as identifying their difficulties and enhancing factors [41].

Our work arose from a broader study which focused on analysing the perception of a sample of pregnant and breastfeeding women in relation to environmental pollutant exposure [42,43,44,45]. Specifically, the objective of this work was to analyse the perception and demands of these pregnant and breastfeeding women concerning their role as a study population, emphasizing the two-way relationship with the research team; this was done to identify the ethical and methodological aspects that needed to be considered in future studies in order to ensure the study population’s active participation and adherence, as well as to improve the transference of the research results in order to promote responsible and participatory research.

## 2. Materials and Methods

### 2.1. Design and Population

This work is based on our experience in a qualitative social study performed in 2015 (Ref. CSO2014-58144-P), that was later expanded with additional funding in 2018 (Ref. AP-0139-2017). Our study aimed to explore the motivations of pregnant and breastfeeding women to avoid persistent toxic compounds in their diet. The criteria for a descriptive and interpretative observational study under the qualitative research paradigm were met following a phenomenological and ethnographic perspective [46,47].

The population was recruited by means of intentional or rational non-probabilistic sampling [48,49]. An equitable and non-discriminatory sample selection was carried out. The inclusion criteria were the following: having been born in Spain, pregnancy at 20-week gestation or, breastfeeding (maternal and/or formula feeding) within a maximum lactation period of six months, and that they belonged to diverse socio-economic strata (Table 1).

The field work was carried out in ten health centres (three hospitals and seven primary care centres) in two Spanish regions, Catalonia (Barcelona and its metropolitan area, Baix Llobregat, Tarragona and Ribera d’Ebre) and Andalusia (Granada and its surroundings, the Valle del Almanzora in Almería, Antequera in Malaga and Cabra in Córdoba).

The fieldwork for the first study began in January 2016, once the corresponding ethics committees gave approval in Catalonia and Andalusia, and ended in September of the same year. All participants were informed of the study objectives and methods, and each gave their informed written consent.

The pregnant and breastfeeding women participating in the two studies received detailed and extensive research information at the start of the interview sessions, focus groups and ethnographies. The participation consent document was separate from the research information document. All participants were first presented with the research information document, which was supplemented by a detailed explanation from the researcher. The participation consent document (Supplementary Material Table S1) and an information document (Supplementary Material Table S2) have been included as supplemental information. The protocols of both the studies were approved by two ethic committees: Ethical committee for clinical research “Parc de Salut Mar” (Ref: 2015/6459/I) and Research Ethics Committee of the Andalusian Health Service in Almería (Ref: 66/2017).

After the sessions, participants’ doubts and concerns regarding exposure to environmental pollutants were solved by the health professionals involved in the study. We acknowledge that this information may stress certain women to some extent. However, this action was taken on the basis of our ethical obligation to inform the population on healthy lifestyle habits and on our belief that the risks would be counterbalanced by the benefits of the information. The two research studies were conducted in accordance with the Helsinki Declaration [50].

### 2.2. Data Collection and Fieldwork Instruments

**Figure 1:**
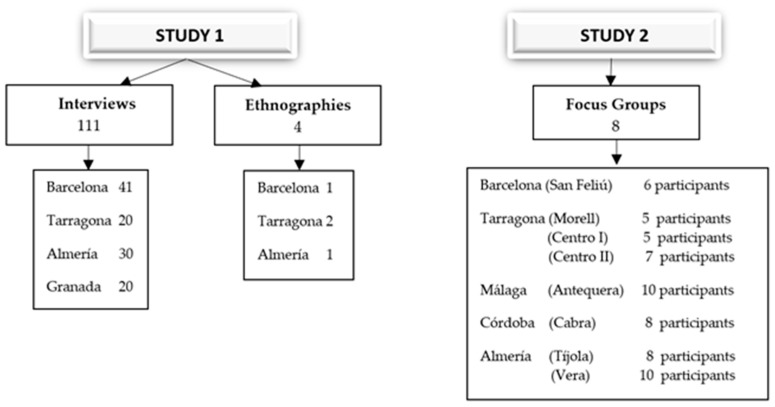
Data collection instruments by field work place.

In an attempt to generate a participatory procedure that would encourage the building of horizontal relationships between participants, we used three different methodological tools for data collection [51,52]. These include 111 semi-structured interviews with the pregnant and breastfeeding women, four targeted ethnographies and eight focus groups as instruments for obtaining primary data (Figure 1).

**Figure 2:**
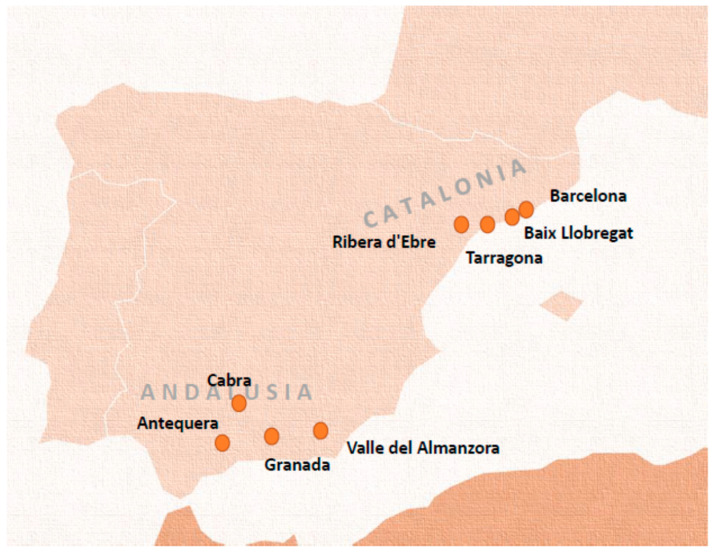
Study areas.

The ethnographies were performed between May 2016 and September 2017 in selected locations from the study regions: a municipality of Baix Llobregat; a neighborhood of the city of Tarragona; two municipalities in Ribera d’Ebre and two municipalities in the Almanzora Valley (Figure 2). With the granting of the second funding, the study was expanded with eight focus groups being held between October 2018 and July 2019.

The semi-structured interviews with the pregnant and breastfeeding women contributed to obtaining data from the women’s discourses with sufficient density and complexity [53,54]. To this end, the interview was structured into five sections addressing the women’s trust and distrust of food, the origin of the food they eat, the preparation of the food they cook, where and with whom they eat and, finally, the women’s knowledge of the toxic compounds persistent in food. This instrument allowed us to understand how the women perceive their relationship to food and the environment, as well as revealing the value systems and standards that underpin these practices [38,39,46]. The questionnaires have been included as supplemental information (Supplementary Material Tables S3 and S4).

The focus groups helped us promote interaction between the women so that they could provide first-hand information. The women’s participation in the groups encouraged them to take part in the discussion and facilitated a flexible and open discourse that enabled intersubjectivity and reflexivity processes. This tool also helped researchers to observe the women’s non-verbal behaviours [46]. In addition, at the end of the session, we presented a guide on recommendations for breastfeeding and pregnant women to avoid potentially-toxic chemical exposures, mainly through food, that had been edited by the research group and it is freely available online [55].

All the interviews and focus groups were conducted in Spanish, and the results were translated into English for publication purposes by a professional translator. The translations were additionally revised by the authors.

The field notes and the in-depth observation during the four ethnographies allowedus to perform a “triangulation” between the methods and data to compare the preferences, standards, social representations and the practices regarding food and health expressed within the different research contexts. During the ethnographies, we accompanied the women in different contexts: at home to observe how they cooked, in the places where they bought food, the places where they ate away from home and with whom they ate. In addition, a comparative approach allowed us to verify how medical standards and recommendations on environmental and nutritional risks are incorporated, modified or ignored by pregnant and breastfeeding women [48].

In our work, we strictly followed current recommendations on qualitative research [47]. In this regard, we sought meaning in the discourse of pregnant and breastfeeding women in a flexible and inductive way, from the particular to the general [48], with the intention of evaluating the perception and demands that pregnant and breastfeeding women had in relation to their role as participants in environmental research studies.

### 2.3. Data Categorization and Analysis

The discourses produced by the pregnant and breastfeeding women were recorded in digital audio format during the interviews and focus groups. In addition, during the focus groups, two of the researchers took notes and observed the participants’ non-verbal language while a third was acting as the moderator. During the ethnographic work, the ethnographers took field notes that were later digitized for computer analysis treatment.

Transcripts of the interviews and focus groups were taken by a professional transcriber. To codify the content of the pregnant and breastfeeding women’s discourses, we relied upon the NVivo 12 software. 

Following the analytical framework for qualitative research recommended by Atkinson and Hammersley [38], Ruíz [39] and Flick [46], we developed an analytical process that helped us to explain the reality of the pregnant and breastfeeding women’s discourses, describe the relationships between the discourses, and synthesize the data into an organized whole. In the coding work, we carried out a synthesis process, grouping different analytical categories that dealt with the same discursive topic.

Following the content analysis, we had the basis to perform an interpretive analysis of the discourse from a hermeneutic perspective [56]. This global vision of the results helped us relate the values and beliefs emanating from the pregnant and breastfeeding women’s discourses with different social theories.

## 3. Results

### 3.1. Research Is of Interest to Pregnant and Breastfeeding Women

In this section, we present participants’ narratives in which they show that the research study theme is also a matter of interest to the pregnant and breastfeeding women.

Although, as researchers, we questioned ourselves while designing the study as to whether the pregnant and breastfeeding women might feel vulnerable addressing the potential contamination of food by persistent toxic substances (PTSs), during the fieldwork they spontaneously stated that research of this type was necessary:

*“No, to add that it seems to me that it’s one of the very important issues and many studies are needed, and this issue needs to be taken seriously because it’s fundamental. Food is basic”*.

(a 37-year-old breastfeeding mother who is an administrative assistant)

Many of the women in our study understand that they may be in a situation of helplessness given the limited studies conducted on pregnancy and lactation in relation to the potential contamination of the body by PTSs over the long term:

*“And I think what is lacking is more studies, that is, more studies to know how these substances can harm us in the long run. But it is true that I believe we are quite helpless”*.

(a 42-year-old breastfeeding mother who is a teacher)

On several occasions the researchers asked if the research topic caused the women discomfort or if they felt uncomfortable reflecting on potential health hazards from possible PTS contamination of food. Most of the women in the study stated that they preferred to have information on persistent toxic substances:

*“No, I haven’t felt uncomfortable, I liked it, it’s always good to be informed and have knowledge. Now, if they tell you... well, now I’m going to get sick from this, what can I do? It’s always good to be informed [...], it’s more interesting than I expected. Really. Yes, it’s an interesting subject”*.

(a 33-year-old pregnant woman who is a salesperson)

For many of the informants, it is important to conduct research that focuses on feeding for the well-being of the mother and baby. The women are aware of the helplessness they suffer, as it is not always possible to determine and know the food circuits.

“I just don’t know to what extent... it can be fixed. Because really, you’re not telling me, “the beach squid...” we don’t know if it is the beach squid or the fruit that comes from certain countries. What fruit?... Is it seasonal?... Strawberries? It’s a bit ambiguous. I think we need to take a closer look. Where it comes from and how it can be channelled. And the producers, I understand, have to be honest about the products’ origins. Nowadays, we know. We know the origin. At my work, in the workers’ restaurant, we know the origin of the products, whether they are first, second, third or fourth rate...”

(a 39-year-old pregnant woman who is a management secretary)

In addition, in one of the focus groups, the women participating asked us to comment on the guide we had drawn up from the results of the first research project. They were very interested to know what the results were and to discuss them with us. Indeed, it is noted that the women found the study interesting not only because it was a source of information on food and environmental toxic substances, but also because of the opportunity we offered them to give their opinion and point out the aspects they agreed or disagreed with. In this sense, the discussion group became an empowerment tool, as the women felt they could co-reflect, co-discuss and co-contribute ideas on an issue that directly affects them, yet which often only the experts decide upon. The discussion group thus became a resistance space against helplessness:

“I don’t trust the market, because there are a lot of harmful things and they don’t put any kind of veto or anything”.

“I do, I do trust it, you see...”

“I solve it by looking at the labels, before I didn’t, but now I do look at what I buy [...] I see if there is anything I don’t know on the labels... When I start to see E-133, E-124, and more and more E’s ... I leave it...”

“Having the information is important... because they trick you. I have a nephew who is now 12 years old and he was diagnosed with obesity and told not to drink packaged juices because perhaps on the label it says no added sugars, 22 calories, for example, and you think, look! Well, it seems okay... but that’s not really what they sell you...”

“Yes, yes, or perhaps they tell you it’s per hundred grams, or per… I don’t know what.... And it seems low but afterwards!!!”

(all from a discussion group, Tarragona)

### 3.2. The Need for Extensive Information on the Research Subject

During the dynamics of the fieldwork, the participants had it explained in detail what persistent toxic substances (PTSs) were. The women took this information as being necessary. Most participants did not know about the presence of toxic substances in food but stressed that it would be important to have this information in order to, at least, be able to choose when buying the product:

“Well, what a bad business, ha ha... sounds very dangerous given the little information we have, for example, this “persistent” thing, and what foods have these “persistents”, how do we know?... Well, I don’t know, maybe the greengrocer’s where I sometimes shop for me, because for my son I go more for organic, but for me, I tell you, we do a fifty-fifty, one day I go to the market and buy the fruit, but who knows, how do I know? And if it’s controlled, shouldn’t it have a label for the user to decide? It’s just that, from what you’re saying, it sounds very ‘heavy’...”

(a 39-year-old pregnant woman who is a nurse)

Only a small number of women reported that they knew what PTSs were and were aware of their potentially harmful health effects:

*“Yes, I’ve heard something about it, because I’ve heard about the relationship between certain substances and infertility, which seems to be that [...] I’ve heard of a study showing that men are now more infertile than they were 40–50 years ago, and they blamed it on food, so I have heard a little in truth”*.

(a 29-year-old pregnant woman who is an instructor)

Some of the women said they trusted the legislation regulating the use of phytosanitary products in Europe, although not in other parts of the world. The idea was also raised that not everything that is local (from the neighbour’s or relative’s garden) is healthier, but that it depends on what is “put on it”:

“ I think it’s true that there are a lot of foods that aren’t desirable, it’s also true that today’s regulations, the legislation, I think has changed a lot and the whole issue is quite controlled... at the phytosanitary level, fertilizers, etc. At least in Europe, that’s why I tell you, I trust Europe, not the rest of the world, and that there are no substances so, many certificates are required that you are not adding heavy metals to the products you sell etc. So I think something will come, it’s clear but, above all, at the pesticide level, but well, I think the minimum and I think our situation today is much better than what existed 40 years ago, I don’t know, the people say, ‘Before it was better and you ate a lot healthier’ Well, if your grandmother had an vegetable plot, maybe, but if not, no, even grandma knows what she puts on it, because nowadays you go to the villages, too, and people do terrible things, they put on products to remove the weeds “Mistol”, and a lot of things that they don’t know how to handle, for me that makes me more afraid than in more industrial production, so what people say, that today we are worse off than quite a few years ago is not true because there were very dangerous fertilizers and phytosanitary products that are now very prohibited, so I believe that in the end there will be things, there are also many degenerative diseases that arise, cancer etc. In the end, our life is very long and there has to be these kinds of things.”

(a 34-year-old pregnant woman who is an agricultural engineer)

Although, as we have pointed out, most women appreciated the information, at the same time, some of the participants expressed discomfort at such recommendations given at such a sensitive life stage. They even related uncertainties when reflecting on possible changes in eating habits in the future:

*“It could be, he he he (laughs), perhaps it will make me look more at the label, it could be. Equally, it might not? I don’t know, I don’t know. I just tell you, maybe because of the pregnancy issue, I don’t want to, but because, I tell you, because the people say, don’t eat this! Don’t eat that! And hey, this yes you can eat! This causes you to, look, I eat what I’ve always eaten and that’s that. So, maybe, perhaps later, when I have the child, if I can do more, let’s see, this thing, what’s in it. That yes, well, because now I have my son, it’s going to be another story. But for me, what I eat is the same to me”*.

(a 39-year-old pregnant woman who is a lawyer)

With the potential vulnerability of the pregnant and breastfeeding women, addressing an issue as sensitive as what they feed themselves and their child, was one of the ethical dilemmas that arose from the beginning. At this vital moment, the pregnant and breastfeeding women make an effort to discern what information can be translated into appropriate food practices. This is how two of the participants expressed themselves:

*“That’s what you have to think about, because if I’m eating plastic and I’m breastfeeding her, I’m passing on the plastic to her in some way and there isn’t much information about it, either. The information is like that, rough. In the sea, there is microplastic in the fish. The fish eat it, then, if you eat the fish, obviously you take in part of it, but there is no more information, and that’s how it is, and you stop to think and you say well, this in the long term, either my kidney or my bladder or my intestine at some point will say enough”*.

(a 37-year-old breastfeeding mother who is a businesswoman)

*“I’ve been told this by my sisters-in-law, for the previous pregnancy. They say, ‘Avoid ham; if you can avoid it, avoid it.’ During the pregnancy, but, I mean, the doctors haven’t told me that, eh? That I shouldn’t eat... I haven’t been told”*.

(a 30-year-old pregnant woman who is unemployed)

### 3.3. Proposals during the Research and Shared Decision-Making

During the first interviews, a topic that emerged that interested the women was the influence of environmental pollution on the possible increase of harmful substances in food. In a shared way, we decided to include it as a topic of discussion for all the interviews and focus groups in the two studies:

*“Totally, it totally has an influence. I, for me, it has a direct influence, because the plants, from the rain, the water that falls, with the contaminated land, are fed from there. And animals that eat contaminated plants, or eat, well, everything... the water, everything, everything, the nitrates, everything that is in the soil, then everything is going to end up in the plant. And we eat all that. So, I do think it affects us. And here in Tarragona, especially. And besides, we don’t know what we’re breathing. I think it affects a lot [...] I had a vegetable garden at home, an urban garden, but I stopped doing it, because I say “I live here, in Ramon y Cajal, and I think I’m eating worse than if I go shopping at the greengrocer’s”, because here there are cars all day long. And, in the end, if I’m wiping away the dirt and that black stuff from the pollution comes out, then that same black stuff has been eaten by the plant. And I stopped doing it, in fact, for that reason. You do not trust the environment”*.

(a 32-year-old pregnant woman who is self-employed)

Another important moment during the research was the offer to bring the concrete requests of the women into our study. On different occasions, after asking them what to recommend for our research, they asked us to explicitly share their interests and give voice to their requests:

Moderator: *“And how would you advise us, what would you say to us, having done this research? If you had to say, well, what is it that you want us to be able to say, what would you wish for?”*

Participant: *“That you make things easier for us, right? That’s what I was saying. If I go to the store, I’m calmer, to say “Good heavens, what I’m buying, I don’t have to look at it and then look again, I don’t have to look at what it costs...”, because there are products that are very ecological, very healthy and such, but then they also cost a lot more money, right? And, sometimes, you can and, sometimes, you can’t. Well, make these two things easier”*.

(a 38-year-old breastfeeding mother who is a teacher)

However, the participants had an even more active attitude. At different points in the research, they reflected on the need to make decisions to minimize the risk of contamination from persistent toxic substances:

*“I think there are solutions, such as trying to consume on an ecological level, so look for companies that work with organic products, or look for livestock cooperatives that work at an ecological level. It seems complicated, but... it is difficult yet not so difficult, because I know of people who only buy organic, organic soap, organic shampoo, which obviously means they have certain resources, what we talked about before, money, it is clear that it is not... but it is possible, having in the house less plastic utensils and more cast iron, glass, but of course, it is a very severe change of mindset”*.

(a 29-year-old pregnant mother who is an instructor)

As intervention strategies, the women propose individual-type actions—having the knowledge to be able to choose—collective—greater awareness of all the parts involved in the process—and protection by health and political authorities to ensure food security:

*“Of course, given that I had no idea about any of this, and I’m caught again, I can’t say anything else, because I have to process it myself, and know and realize and try to be more careful when buying and where to buy it, if it’s packaged or if it’s loose, and control a little more because they had no idea”*.

(a 30-year-old pregnant woman who is an office worker)

“Pfff.... At the collective level, it would be to raise awareness among all those who provide us with food, livestock, the... well, in the end, do you know what’s going on? Because in society we are so irresponsible, we are destroying ourselves, in that sense, because if we looked after the environment (...), if the people who have to provide us with food, instead of using pesticides, they used natural fertilizers, if they became more aware, it would surely change. But since it isn’t like that, they need things to come out fast, to make the greatest quantity to be able to make a profit, and we don’t achieve this. (...) and on an individual level, I think it’s looking for things that are more natural, but it’s complicated. You have to know that it is from a person who is growing their vegetables with natural fertilizers, that they don’t put anything else on, that they also normally have the natural ones that don’t have additives, very soon they go bad, it is a little like trying to look, that you don’t always have the ability, nor do you have it at hand.”

(a 38-year-old pregnant woman who is a nursing auxiliary)

### 3.4. Availability to the Women of the Information Coming from the Research

At the end of our first study, the guide was presented to the pregnant and breastfeeding women participating in the research. Sessions were conducted in Catalonia and Andalusia. Subsequently, during the focus groups in the second study, some women who had attended these briefings participated. A participant in the Antequera group expressed how having the results of our work had influenced her habits when selecting food while shopping:

*“I always had notions of what is healthy, more or less, because in my house my parents have instructed us a lot about such a diet, how it is, the Mediterranean diet and all that. Now during the pregnancy, I am aware of the foods that are harmful, because look, it all started because you gave us the study that you did in Antequera hospital, she passed us the information, I read it and I said: <Wow, this I really have to look at>. And then you start looking at it”*.

(a 35-year-old pregnant woman who is a dentist)

At another of the briefings on our work held in the Almanzora Valley in the municipality of Albox (Andalusia), one participant explained how reading the guide had made her reflect on the plastic pollution affecting the sea:

*“And especially if you stop to read your information and start thinking about pollution above all in the water, which says we’re eating plastics, and you’re surprised like: how is it I’m eating plastic?”*.

(a 32-years-old breastfeeding mother who is a psychologist)

This same idea, and the sense of helplessness, also appeared in the accounts of women in Catalonia.

*“It seems that we are condemned to disease (....) to see what comes out in the study, because I think we are all concerned with the chemical issue, even if we continue with the same habits, because you can improve the habits and go shopping in ecological shops, but you can’t buy everything organic and if you live in Barcelona, you have chemicals everywhere. To see what you come out with, because it’s frightening, now you also hear a lot about cancer lately, which I think has a lot to do with food and food production habits”*.

(a 38-year-old breastfeeding mother who is a social worker)

“What you carry, you can pass on to your baby, what circulates through you when he is in-utero is connected to you, to your circulatory system, and if you breastfeed, what you’re consuming is being passed to him. If you consume stimulant drinks, you pass on the stimulant drinks to him, if you consume things with a lot... that are very gassy, you pass the gases to him, yes”.

(a 39-year-old pregnant woman who is a nursing auxiliary)

## 4. Discussion

In this research, we evidenced the need of pregnant and breastfeeding women for more detailed and accurate information on the risk of environmental pollutant exposure on their crucial life stage. In addition, these women claimed for a more participatory role in research studies

These issues go beyond the results of a research project themselves, as they involve a two-way relationship between researchers and participants, which involves the implementation of actions and the co-creation of an ethical commitment (beyond informed consent) to research.

As Bourdieu points out, science is not neutral and is based on framed ideals that make sense within the society where it develops; that is, it could be said that the field of science is crossed by interests, hierarchical positions and power relationships that lead to the imposition of certain epistemological and theoretical perspectives that shape dominant scientific morals [56]. Considering, moreover, that ethics are a reflection on morality [57], which provides critical patterns for the prosecution of the society’s actions, it makes sense to think that any research must be subject to ethical parameters dependent on the socio-historical context that determines it [58].

There is now growing concern about social justice issues [59]. Currently, this “social justice” refers to how goods and burdens are distributed in society [60], the definition of people’s rights and how decision-making processes are carried out [61]. Among the goods that are distributed are income and wealth, power and opportunities, obligations and rights, as well as jobs and honours [62].

For this reason, precisely because every scientific process is channelled by the hegemonic principles legitimized by each society, and by the characteristics inherent in qualitative research, we contemplate conducting responsible research with others and with nature, as well as research committed to transformation and social justice. To do this, we had to recognize the researcher as a moral subject and his/her research practice as a moral action. As Peñaranda (2015) [62] points out, qualitative research must be ethically thought out and oriented, and cannot be thought or raised without the participation of the beneficiaries, because only their participation guarantees their empowerment and, in the specific case of this research, the right of women to decide and take control over their role in environmental research studies in a period as vitally important as pregnancy and lactation.

Different authors [63] state that women, regardless of their socio-economic status, experience moments of distress regarding the development and care of their future child during pregnancy and breastfeeding. We agree with these authors [64] when they say that the focus of their concerns shifts from topic to topic as they find answers to their uncertainties, so did the women in our studies when they recounted the lack of information on feeding during pregnancy and breastfeeding.

Although feeding in the pregnancy and breastfeeding period is an issue addressed by health professionals in Spain during maternal education, women obtain the information they need from other sources such as friends, books, the internet and mainly from their mothers. In our studies, we have observed that food information promoted from the healthcare sector did not always meet its expectations, being scant or lacking, and particularly when it came to addressing potential chemical contamination through food or the environment [42].

Not transmitting extensive information on potential risks in pregnancy and lactation has been a common practice in the care activity of health workers [65]. It has also been a common decision not to have pregnant and breastfeeding women as a study population in research work, as they are considered a vulnerable population, and for this reason underrepresented in scientific studies [23].

The issues above were an ethical dilemma when designing our research work. Would the research subject be useful for women, or would it be a source of distress, by stressing potential food and environmental risks during such a sensitive life period? In our research work, after analysing the narratives of the pregnant and breastfeeding women, most said they want to have more information about the potential risks of chemical contamination through food and the environment. On the other hand, women are distressed by the uncertainty resulting from the lack of information about these risks while showing feelings of helplessness over the lack of alternatives to avoid these contaminants when consuming food or in their relationship to the environment. Indeed, most women prefer to be a part of the advancing knowledge on these issues and have the opportunity to choose in a vital process that involves their own body and person, rather than not to have all the information or have other social agents (institutions, health workers, the food industry...) decide for them.

Van der Zande et al. [22] point out that the concept of a ‘vulnerable’ population in research is used to specify populations whose ability to make decisions about participation in studies is somehow compromised by their limited cognitive ability. For this reason, we agree with these authors that such a designation for pregnant and breastfeeding women is inappropriate and disrespectful. During our research work, we were especially careful to extensively inform participants, but the reason was not because we felt that pregnant and breastfeeding women are a vulnerable population for research [66]. The opposite is the case, giving extensive information to participants was aimed at empowering women to make an independent decision as to whether they participate or not in our studies.

We concur with Krubiner and Faden [23] that there is nothing about the state of pregnancy and breastfeeding that makes women unable to give consent or reject research. Moreover, instead of protecting the health interests of pregnant women and their children, this designation as a vulnerable population has had the opposite effect. It has contributed to the exclusion of pregnant women from research activities, which in itself is pernicious to the health of pregnant women [22]. In our studies, the female participants encourage scientists to conduct more research into chemical contamination risks. The women did not feel vulnerable and freely made the decision whether to participate or not in the research. Of the women initially recruited, only two refused to participate, a pregnant woman who had health problems during pregnancy and another breastfeeding mother who said she had time problems connected to balancing her work and family, both in the second research study.

In designing our studies, we set out not to make pregnant and breastfeeding women passive research subjects [26]. The qualitative research techniques used, such as in-depth interviews, focus groups and ethnographies, gave the women the opportunity to widely recount their perceptions about the risk of chemical contamination in food and in the environment [46]. But beyond ethically promoting inclusive research, we managed to get women to have an active voice by participating in shared decision-making regarding the research [67,68]. Since these qualitative techniques, in addition to relating the women’s opinions about products they consider toxic or not (*what*), have also given us the opportunity to know *why*, that is, the socio-cultural factors that explain their ways of understanding food risk; and this can help us to design much more effective public health prevention policies. As we noted in the introduction, and in accordance with Rodríguez (2009) [37], the narratives have allowed us to know how food is lived and interpreted, and what are the main support needs of women and those they care for. In this sense, narratives promote empowerment in developing food care and self-care capacities, resources and skills for pregnant and breastfeeding women, as well as identifying the difficulties and enhancing factors [42,43,44,69,70].

After the initial fieldwork, the participants’ concerns emerged regarding the direct health impact that environmental pollution could have on them, and the indirect impact regarding the contamination of the food they eat. In response to their information needs on this topic, we decided to include issues on environmental pollution in a shared way so that the participants could express their opinions in the interviews, focus groups and ethnographies. We agree with various authors [71] when they state that participants in research work should play an active and empowered role in the research by encouraging shared decision-making during the process, especially in research with pregnant and breastfeeding women [72]. This empowerment to which we refer is not limited to the individual dimension but takes on a multidimensional nature by promoting the creation of a dialogue that ensures that their discourse plays an active role in designing health education strategies for pregnant and breastfeeding women. We agree with Lyons et al. (2013) [71] that involving participants in decisions regarding the communication and implementation of research results is a matter of equity and social justice.

Despite our study aimed to capture information from a wide population, our study population had predominantly higher studies, ranging from 30 to 39 years old. This might undermine the extrapolation of our findings to lower social classes and younger age ranges, so that further research is warranted on these groups. However, it is noteworthy that our age group represents approximately 65% of newborns in Spain [73]. In addition, we recruited women with >20 weeks of gestation, whose perceptions might be different to those with <20 weeks. Lastly, our limited sample size hampered comparisons between pregnant and breastfeeding women, which might be a very interesting topic to address in further researches.

## 5. Conclusions

Pregnant and breastfeeding women demand to participate more frequently in environmental research studies and do not consider themselves a vulnerable population for research. Beyond their participation, the women encourage researchers to count on them in shared decision-making before, during and after the studies. Pregnant and breastfeeding women in Spain ask for greater interaction with researchers and propose a dialogical relationship between valid partners. The participants in our studies do not want to simply be informants, rather they want to be active and empowered members throughout the research process.

Knowing the perceptions and demands of our participants regarding their role in environmental research studies has significant value from the public health practice and research standpoint. The active voice of women in environmental research studies will help promote public health policies for this population sector that, benefitting from the voices of their protagonists/beneficiaries, will have a greater guarantee of impact and success.

We conclude that our pregnant and breastfeeding women claim more research focused on their collective, as well as clearer, more accessible and structured information on the risks of exposure to environmental contaminants. In addition, they do not want to simply be informants; rather, they ask to be active and empowered members by providing their opinions and arguments throughout the research process.

## Figures and Tables

**Table 1:** Characteristics of the pregnant and breastfeeding women participants.

Participants 173	84	89
Age range		
Age-20–29	12	16
Age-30–39	65	64
Age-40+	7	9
Education Level		
Primary	6	4
Secondary	27	29
Higher	51	56
Number of children		
1 Child	47	54
2 children	29	31
3 Children or +	8	4
Province		
Almería	24	24
Barcelona	25	32
Córdoba	5	3
Granada	9	11
Málaga	6	4
Tarragona	16	14
Autonomous Community		
Andalucía	44	42
Cataluña	41	46

## Supplementary Materials

The following are available online at https://www.mdpi.com/article/10.3390/ijerph18084149/s1, Table S1: CONSENTIMIENTO INFORMADO DEL PARTICIPANTE. Table S2: DOCUMENTO DE INFORMACIÓN AL PARTICIPANTE. Table S3: ENCUESTA NUTRICIONAL EN SITUACIÓN DE EMBARAZO. Table S4: ENCUESTA NUTRICIONAL EN SITUACIÓN DE LACTANCIA.
