# Supplementary material for: Perception and Demands of Pregnant and Breastfeeding Women Regarding Their Role as Participants in Environmental Research Studies
Source: Int J Environ Res Public Health. 2021 Apr 14;18(8):4149. doi: 10.3390/ijerph18084149 (PMC8070940; doi:10.3390/ijerph18084149)
Supplement: Supplementary file 1 [file ijerph-18-04149-s001.zip › ijerph-1178526-supplementary/ijerph-1178526 -Suppmentary-Table S1.pdf]

ESTE DOCUMENTO SE FIRMARÁ POR DUPLICADO QUEDÁNDOSE
UNA COPIA EL INVESTIGADOR Y OTRA EL PARTICIPANTE

# Table S1: CONSENTIMIENTO INFORMADO DEL PARTICIPANTE

**PROYECTO: "Percepción del riesgo de contaminación por CTP a través de la alimentación en mujeres embarazadas y lactantes: Estudio MUMFOOD"**

Yo, *(nombre y apellidos)*……………………………………………………………………..,

☐ He leído la hoja de información que se me ha entregado.

☐ He sido informada de manera clara de los objetivos de la investigación y he recibido suficiente información.

☐ He sido informada que la reunión del grupo tendrá una duración de unos 60 minutos.

☐ He hablado con: *(nombre del investigador)*…............................................

☐ He podido hacer preguntas sobre el estudio y he sido informada que cualquier otra pregunta que tenga en relación con el estudio será respondida oportunamente por teléfono o correo electrónico.

☐ Comprendo que mi participación es voluntaria.

☐ Comprendo que si algún tema de conversación durante el desarrollo de la reunión del grupo me parecen incómodo, tengo el derecho de hacérselo saber al investigador o de no participar.

☐ Comprendo que puedo retirarme del estudio cuando quiera y sin tener que dar explicaciones.

☐ He sido informada que la reunión de grupo será grabada y que una vez trascrita la grabación será destruida.

☐ He sido informada que la información que provea en el curso de esta investigación es estrictamente confidencial y no será usada para ningún otro propósito fuera de los de este estudio sin mi consentimiento.

☐ He sido informada de que mis datos personales serán protegidos de acuerdo con lo dispuesto en la Ley Orgánica 15/1999 de Protección de Datos de Carácter Personal y serán utilizados únicamente con los fines especificados en la hoja de información.

☐ Comprendo que tengo los derechos de acceso, rectificación, cancelación y oposición a mis datos de carácter personal de acuerdo con lo dispuesto en la Ley Orgánica 15/1999 de Protección de Datos de Carácter Personal.

☐ He sido informada que puedo recibir información sobre los resultados del estudio cuando éste haya concluido, si así lo solicito.

☐ Presto libremente mi conformidad para participar en el estudio y doy mi consentimiento para el acceso y utilización de mis datos en las condiciones detalladas en la hoja de información.

☐ Entiendo que una copia firmada de este Consentimiento Informado me será entregada.

***Firma del participante:***

Nombre:
Fecha:

***Firma del investigador:***

Nombre:
Fecha:
