# Supplementary material for: Perception and Demands of Pregnant and Breastfeeding Women Regarding Their Role as Participants in Environmental Research Studies
Source: Int J Environ Res Public Health. 2021 Apr 14;18(8):4149. doi: 10.3390/ijerph18084149 (PMC8070940; doi:10.3390/ijerph18084149)
Supplement: Supplementary file 1 [file ijerph-18-04149-s001.zip › ijerph-1178526-supplementary/ijerph-1178526-Suppmentary-Table S2.pdf]

Table S2: Documento de información al participante

Apreciada Sra.:

El proyecto "Percepción del riesgo de contaminación por CTP a través de la alimentación en mujeres embarazadas y lactantes: Estudio MUMFOOD" es un estudio interdisciplinario adscrito al Servicio Andaluz de Salud y al *Departamento de Antropología Social* de la *Universidad de Barcelona*.

El proyecto se centra en el estudio de la confianza/desconfianza en el consumo de alimentos percibida por los actores sociales preocupados por la presencia de sustancias tóxicas (Compuestos Tóxicos Persistentes -CTP-) y las responsabilidades atribuidas sobre los posibles efectos que estas sustancias acarrean o podrían producir a largo plazo a la salud humana. La contaminación interna que deriva del impacto de estas sustancias tiene importantes implicaciones culturales, sociales, ideológicas y económicas que este proyecto pretende analizar en el universo de las mujeres embarazadas y lactantes, por ser este un período del ciclo vital en el que la alimentación constituye un tema central en la salud de la mujer.

Para nuestro proyecto y el estudio de esta temática, sería de gran interés poder contar con su participación mediante una reunión de grupo entre 8-10 mujeres embarazadas o lactantes. La participación en este estudio es estrictamente voluntaria y puede retirarse del proyecto en cualquier momento. Si usted accede a participar, se le pedirá conversar en grupo los temas anteriormente citados, aproximadamente durante unos 60 minutos de su tiempo. Si algunas de los temas durante el desarrollo de la reunión del grupo le parecen incómodas, tiene usted el derecho de hacérselo saber al investigador o de no mostrar su opinión.

La reunión del grupo será grabada, de modo que el investigador pueda transcribir después las ideas que usted haya expresado. Una vez transcritas las conversaciones acontecidas en el grupo, las grabaciones se destruirán. Sus opiniones expresadas en el desarrollo de la reunión del grupo serán codificadas usando un número de identificación, por lo que éstas serán anónimas, y la información que se recoja será confidencial y no se usará para ningún otro propósito fuera de los del estudio. De esta manera, teniendo en cuenta los principios éticos que debe tener una investigación de estas características, se garantiza estrictamente el anonimato y la confidencialidad de los datos, siempre asegurando el riguroso cumplimiento del secreto profesional en el uso y manejo de la información y el material obtenidos.

Los datos tendrán como finalidad ser utilizados para la investigación, guardándose siempre sus datos personales en un lugar seguro de tal manera que ninguna persona ajena pueda acceder a esta información y atendiendo a un estricto cumplimiento de la *Ley Orgánica 15/1999 sobre la Protección de Datos de Carácter Personal (LOPD)* y el articulo 3, punto 6 del *Real Decreto 223/2004*. También puede ejercer los derechos de acceso,

rectificación, cancelación y oposición previstos por la *LOPD* y su petición será atendida de manera inmediata.

Así mismo, se garantiza que, siguiendo los principios de la *Declaración de Helsinki*, la investigación estará sujeta a una revisión ética, se basará en un conocimiento científico exhaustivo y estará conducida por investigadores expertos.

Si desea conocer los resultados del proyecto, puede solicitarlo durante el desarrollo del grupo o en cualquier otro momento y se los haremos llegar una vez haya concluido el estudio.

Agradecemos de antemano su colaboración y quedamos a su disposición para resolver cualquier tipo de duda o aclaración. Puede dirigirse a Miguel Company (miguel.company.sspa@juntadeandalucia.es / miguelcompanymorales@gmail.com), investigador principal del proyecto mediante correo electrónico o telefónicamente (950429541).

Atentamente,

Dr. Miguel Company Morales
Investigador principal
“Estudio MumFood”
