# Supplementary material for: Perception and Demands of Pregnant and Breastfeeding Women Regarding Their Role as Participants in Environmental Research Studies
Source: Int J Environ Res Public Health. 2021 Apr 14;18(8):4149. doi: 10.3390/ijerph18084149 (PMC8070940; doi:10.3390/ijerph18084149)
Supplement: Supplementary file 1 [file ijerph-18-04149-s001.zip › ijerph-1178526-supplementary/ijerph-1178526-Suppmentary-Table S3.pdf]

CONSEJO GENERAL DE COLEGIOS OFICIALES DE FARMACÉUTICOS

Table S3: ENCUESTA NUTRICIONAL EN SITUACIÓN DE EMBARAZO

DATOS GENERALES

Edad de la mujer: _________años **Altura:** _________cm **Peso pre-gestacional:**__________kg

Peso actual de la mujer: _________kg **Circunferencia del brazo:** _________cm

Nº hijos nacidos: ☐ 0 ☐ 1 ☐ 2 ☐ 3 ó más

Nivel de estudios: ☐ Sin estudios ☐ Primarios ☐Bachillerato o F.P. ☐ Titulación universitaria

¿Cómo considera que es su salud actual comparada con la de otras mujeres en su situación?

☐ Muy buena ☐ Buena ☐ Regular ☐ Mala ☐ Muy mala ☐ NS/NC

¿Cómo considera que es su alimentación actual comparada con la de otras mujeres en su situación?

☐ Equilibrada ☐ Bastante equilibrada ☐ Poco equilibrada ☐ Muy desequilibrada ☐ NS/NC

Actividad física en un día típico (horas totales):

Horas tumbada o dormida: _________

Horas de actividades sentada: _________

Horas de actividades de pie o en movimiento: _________

INFORMACIÓN OBSTÉTRICA

Tipo de embarazo: ☐ Único ☐ Gemelar ☐ Triple o más **Semana de gestación:** _________semanas (2-42)

Paridad: ☐ 1º embarazo ☐ 2º embarazo ☐ 3º embarazo o más **Proximidad entre embarazos:** ☐ <1 año ☐ ≥ 1 año

Abortos previos: ☐ 0 ☐ 1 ☐ >1 **Antecedentes de bajo peso al nacer:** ☐ Sí ☐ No

INFORMACIÓN DE LA LACTANCIA

Tipo de lactancia prevista (meses):

☐ Materna 0-3 m ☐Materna 0-6 m ☐Artificial 0-6 m ☐Mixta 0-3 m ☐ Mixta 0-6 m

HÁBITOS

Tabaquismo: ☐ No ☐ Previo ☐ Activo ☐ Pasivo

Consumo de alcohol: ☐ No ☐ Previo ☐ Puntual ☐ Frecuente

Consumo de drogas: ☐ No ☐ Previo ☐ Puntual ☐ Frecuente

ESTADO FISIOPATOLÓGICO

Diabetes gestacional actual (con diagnóstico médico confirmado): ☐ Sí ☐ No

Enfermedades de la mujer: ☐ Problemas leves ☐ Una enfermedad grave ☐ Más de una enfermedad grave

Dietas especiales: ☐ Hipocalórica ☐ Hipoglucídica ☐ Hipolipídica ☐Hiposódica
☐ Vegetariana estricta ☐ Ovolácteovegetariana ☐ Otras ☐ Ninguna

HÁBITOS ALIMENTARIOS

Frecuencias de consumo: nunca (0) / mensual (1, 2 ó 3) /semana (1, 2, 3, 4, 5 ó 6) /diario (1, 2, 3, 4, 5, 6 ó más)

Lácteos: ___________ **Frutas:** ___________

Huevos: ___________ **Frutos secos:** ___________

Carnes: ___________ **Legumbres:** ___________

Embutidos y fiambres: ___________ **Pan:** ___________

Pescados: ___________ **Arroz, pasta y patatas:** ___________

Ensaladas y verduras: ___________ **Bollería y repostería industrial:** ___________

Suplementación de la dieta:

☐ Leche enriquecida en calcio/vitaminas ☐ Fibra/Prebióticos ☐ Probióticos ☐ Yodo/Sal yodada

☐ Ácido fólico/Vitamina B_{12} ☐ Hierro ☐ Polivitamínicos y minerales ☐ Ninguno

Tipo de grasa más utilizada para aliñar/cocinar:

☐ Oliva virgen ☐ Oliva ☐ Girasol ☐ Mantequilla ☐ Margarina

Tipo de edulcorante más utilizado para endulzar alimentos/ bebidas:

☐Azúcar ☐ Miel ☐ Fructosa ☐ Sacarina ☐ Ninguno
