# Supplementary material for: Perception and Demands of Pregnant and Breastfeeding Women Regarding Their Role as Participants in Environmental Research Studies
Source: Int J Environ Res Public Health. 2021 Apr 14;18(8):4149. doi: 10.3390/ijerph18084149 (PMC8070940; doi:10.3390/ijerph18084149)
Supplement: Supplementary file 1 [file ijerph-18-04149-s001.zip › ijerph-1178526-supplementary/ijerph-1178526-Suppmentary-Table S4.pdf]

Table S4: ENCUESTA NUTRICIONAL EN SITUACIÓN DE LACTANCIA

DATOS GENERALES

Edad de la mujer: ________años **Altura:** ________cm **Peso pre-gestacional:**__________kg

Peso en el posparto:_________kg **Peso actual de la mujer:**________kg **Circunferencia del brazo:**__________cm

Nº hijos nacidos: ☐ 1 ☐ 2 ☐ 3 ó más

Nivel de estudios: ☐ Sin estudios ☐ Primarios ☐Bachillerato o F.P. ☐ Titulación universitaria

¿Cómo considera que es su salud actual comparada con la de otras mujeres en su situación?

☐ Muy buena ☐ Buena ☐ Regular ☐ Mala ☐ Muy mala ☐ NS/NC

¿Cómo considera que es su alimentación actual comparada con la de otras mujeres en su situación?

☐ Equilibrada ☐ Bastante equilibrada ☐ Poco equilibrada ☐ Muy desequilibrada ☐ NS/NC

Actividad física en un día típico (horas totales):

Horas tumbada o dormida: ________

Horas de actividades sentada: ________

Horas de actividades de pie o en movimiento: ________

INFORMACIÓN OBSTÉTRICA

Abortos previos: ☐ 0 ☐ 1 ☐ >1 **Antecedentes de bajo peso al nacer:** ☐ Sí ☐ No

INFORMACIÓN DE LA LACTANCIA

Tipo de lactancia actual (meses):

☐ Materna 0-3 m ☐Materna 0-6 m ☐Artificial 0-6 m ☐Mixta 0-3 m ☐ Mixta 0-6 m

Edad del lactante: ________semanas (1,2, 3, 4) /_________meses (1, 2, 3, 4, 5, 6, 7, 8, 9, 10, 11, 12)

Sexo del lactante: ☐ Varón ☐ Mujer **Peso del niño al nacer:** ________kg **Peso actual del niño:** ________kg

HÁBITOS

Tabaquismo: ☐ No ☐ Previo ☐ Activo ☐ Pasivo

Consumo de alcohol: ☐ No ☐ Previo ☐ Puntual ☐ Frecuente

Consumo de drogas: ☐ No ☐ Previo ☐ Puntual ☐ Frecuente

ESTADO FISIOPATOLÓGICO

Antecedentes de diabetes gestacional (con diagnóstico médico confirmado): ☐ Sí ☐ No

Dietas especiales: ☐ Hipocalórica ☐ Hipoglucídica ☐ Hipolipídica ☐Hiposódica
☐ Vegetariana estricta ☐ Ovolácteovegetariana ☐ Otras ☐ Ninguna

HÁBITOS ALIMENTARIOS

Frecuencias de consumo: nunca (0) / mensual (1, 2 ó 3) /semana (1, 2, 3, 4, 5 ó 6) /diario (1, 2, 3, 4, 5, 6 ó más)

**Lácteos:**	___________	**Frutas:**	___________
**Huevos:**	___________	**Frutos secos:**	___________
**Carnes:**	___________	**Legumbres:**	___________
**Embutidos y fiambres:**	___________	**Pan:**	___________
**Pescados:**	___________	**Arroz, pasta y patatas:**	___________
**Ensaladas y verduras:**	___________	**Bollería y repostería industrial:**	___________

Suplementación de la dieta:

☐ Leche enriquecida en calcio/vitaminas ☐ Fibra/Prebióticos ☐ Probióticos ☐ Yodo/Sal yodada
☐ Ácido fólico/Vitamina B_{12} ☐ Hierro ☐ Polivitamínicos y minerales ☐ Ninguno

Tipo de grasa más utilizada para aliñar/cocinar:

☐ Oliva virgen ☐ Oliva ☐ Girasol ☐ Mantequilla ☐ Margarina

Tipo de edulcorante más utilizado para endulzar alimentos/ bebidas:

☐Azúcar ☐ Miel ☐ Fructosa ☐ Sacarina ☐ Ninguno
